# Plasma and Urinary Levels of Nerve Growth Factor Are Elevated in Primary Hypertension

**DOI:** 10.1155/2022/3003269

**Published:** 2022-03-01

**Authors:** Fumihiro Tomoda, Atsumi Nitta, Hiroko Sugimori, Tsutomu Koike, Koichiro Kinugawa

**Affiliations:** ^1^The Faculty of Health Science, Fukui Health Science University, Fukui, Japan; ^2^The Second Department of Internal Medicine, University of Toyama, Toyama, Japan; ^3^The Department of Pharmaceutical Therapy & Neuropharmacology, University of Toyama, Toyama, Japan

## Abstract

Nerve growth factor (NGF) is the main neurotrophic factor that can control sympathetic nerve innervation and sympathetic neural activity in cardiovascular organs. Although NGF overproduction and its influences on the sympathetic nervous system have been shown in hypertensive animals, NGF status and its association with sympathetic nerve activity have not yet been explored in human hypertension. In the present study, therefore, plasma and urinary levels of NGF and those of catecholamines (i.e., indices for NGF status and sympathoadrenal activity, respectively) were compared between 83 untreated primary hypertensives without apparent cardiovascular damages and 81 healthy normotensive subjects. Plasma and urinary levels of NGF were significantly greater in the hypertensive group (311 ± 158 pg/mL and 72.7 ± 54.0 ng/g of Cr) than in the normotensive group (168 ± 188 pg/mL and 54.5 ± 38.8 ng/g of Cr) (*p* < 0.05 for each measurement), even if the baseline differences of age and gender between the groups were adjusted. Similarly, plasma and urinary levels of catecholamines were significantly higher in the hypertensive group than in the normotensive group except for plasma noradrenaline. In addition, despite no significant correlations between plasma levels of NGF and catecholamines in both groups, urinary NGF significantly correlated positively with both urinary noradrenaline and urinary adrenaline in the hypertensive group (*r* = 0.259, *p*=0.018 and *r* = 0.232, *p*=0.035), but not in the normotensive group (*r* = 0.115, *p*=0.307 and *r* = −0.018, *p*=0.871). On the contrary, plasma and urinary levels of NGF as well as those of catecholamines did not associate with any systemic hemodynamic indices such as blood pressure and pulse rate in either group. Thus, primary hypertension was characterized by the enhancements of both NGF status and sympathoadrenal activity and the positive relationship between them. Our data indicate that enhanced NGF status and subsequent NGF-induced sympathoadrenal overactivity could occur in primary hypertension.

## 1. Introduction

It has been proposed that increased sympathetic neural activity could be involved in the initiation and development of hypertension [1–4]. Sympathetic overactivity could also contribute to the onset of hypertension-related cardiovascular structural changes, such as left ventricular hypertrophy and vascular hypertrophy [5, 6]. However, the precise cause of sympathetic overactivation remains undefined in hypertension, although some abnormalities in the modulation of the sympathetic nervous system by the central nervous system, cardiovascular reflex, or humoral metabolic factors have been postulated as the possible candidates [1, 2].

Nerve growth factor (NGF), a member of the neurotrophin family, is a protein required for the growth and maintenance of peripheral sympathetic neurons and neural crest-derived sensory neurons [7–9]. NGF is synthesized and released in organs innervated by sympathetic postganglionic neurons [7, 8]. Thereafter, NGF is incorporated by the nerve endings of postganglionic neurons and acts at their neural cell bodies to control sympathetic innervation and sympathetic neural activity into the organs [7, 8]. In addition, brain-derived neurotrophic factor, another member of the neurotrophin family, could also affect baroreflex function in the central nervous system to alter sympathetic outflow of preganglionic neurons [7, 10]. Thus, in the sympathetic innervated organs, sympathetic neural influence could be regulated by their own production of NGF as well as the influences of NGF on both preganglionic and postganglionic neurons. Therefore, it could be hypothesized that abnormal synthesis and/or secretion of NGF could enhance sympathetic neural activity, potentially leading to the occurrences of hypertension and hypertensive cardiovascular damages. This hypothesis is supported by the observation that NGF mRNA and/or protein increase in the sympathetic innervated organs such as the blood vessels, heart, and kidneys of spontaneously hypertensive rats (SHRs), an animal model of human primary hypertension [11–14]. Conversely, an administration of antiserum against NGF attenuates or prevents the development of hypertension and vascular hypertrophy concomitantly with reductions in sympathetic innervation and sympathetic neural activity in SHRs [13–16]. However, NGF status and its association with sympathetic nerve activity have not yet been explored in human hypertension. In the present study, therefore, plasma and urinary levels of NGF and those of catecholamines (i.e., indices for NGF status and sympathoadrenal activity, respectively) were compared between untreated primary hypertensives without apparent cardiovascular damages and healthy normotensive subjects.

## 2. Methods

### 2.1. Study Population

The study design was approved by the Ethics Committee at University of Toyama. Eighty-three untreated outpatients with primary hypertension and 81 normotensive subjects were consecutively enrolled into the study after obtaining informed consent at the 2nd Department of Internal Medicine at Toyama University Hospital or the Health Care Center affiliated to Hokuriku Electric Power Company. All subjects completed a screening history and physical and laboratory examinations. Blood pressure and pulse rate were determined using a sphygmomanometer on three separate visits over a 4-week period. On each occasion, 3 consecutive measurements of blood pressure and pulse rate were performed by the physicians 30 to 60 seconds apart after 5 minutes of rest in the sitting position, and mean values of those readings were calculated. In the individual cases, the averages of the mean values at three different visits were adopted as their blood pressure and pulse rate. The diagnosis of hypertension was made on the basis of diastolic blood pressure more than 90 mmHg or systolic blood pressure more than 140 mmHg. In contrast, normotensive subjects were confirmed to have blood pressure less than 140/90 mmHg. In the hypertensive group, patients with macroalbuminuria (i.e., urinary albumin >300 mg/g of Cr), impaired renal function (i.e., estimated glomerular filtration rate (eGFR) < 60 mL/min/1.73 m^2^), secondary hypertension, diabetes mellitus, or clinically evident cardiovascular diseases were excluded by laboratory analysis and/or radiological imaging as clinically indicated. The normotensive subjects were also judged to be healthy on physical and laboratory examinations and to have no family history of hypertension according to their medical history. All the studied subjects had never taken any medications affecting blood pressure and sympathetic nerve activity.

### 2.2. Study Protocol

The subjects were instructed not to take food, alcohol, caffeine, and cigarettes within 12 hours before the study. After an overnight fast, each subject was requested to visit our institutes in the morning and urinate to collect the second or third morning urine. Then, a venous catheter was inserted into the antecubital vein, and following 30 min supine rest, venous blood was collected gently without vein occlusion.

### 2.3. Measurements of NGF and Catecholamines

For measurement of plasma levels of NGF and catecholamines, blood samples were collected into EDTA tubes on ice and centrifuged in a refrigerated centrifuge to get the plasma. For measurement of urinary levels of NGF and catecholamines, urinary samples were collected in polyethylene bottles containing 100 *μ*L of 0.6 M hydrochloric acid. Both plasma and urinary samples were kept in the refrigerator at −20°C until the assay. Plasma and urinary levels of NGF were measured using commercial ELISA kits (R&D, USA) [17]. This assay system is specific for the measurement of human beta nerve growth factor and has less than 1% cross-reactivity against other neurotrophic factors such as human brain-derived neurotrophic factor, neurotrophin 3, and neurotrophin 4/5. The manufacturer's description about NGF assay reveals that the detection range is from 62.5 to 4000 pg/mL and the intra-assay coefficients of variation are 7.2%. The additional recovery rate against 500 pg/mL was over 90%. The assays for NGF were performed according to the manufacturer's instruction, and the sample concentrations in each plate were calculated according to standard curves and dilution factors. Plasma and urinary levels of both noradrenaline and adrenaline were determined by high-performance liquid chromatography and electrochemical detection. Urinary albumin concentration was determined using commercial ELISA kits (ASSAYPRO, USA). Serum and urinary levels of creatinine were measured with an enzymatic method. Other biochemical parameters were measured using conventional laboratory techniques.

### 2.4. Statistical Analysis

Data for continuous variables are presented as mean ± standard deviation (SD). Categorical variables are expressed as numbers. Urinary levels of NGF, catecholamines, or albumin were expressed as the urinary concentration ratio of each actual value to creatinine. The body mass index (BMI) was calculated as body weight (kg) divided by height squared (m^2^). The effective glomerular filtration rate (eGFR) was obtained using the following formula: eGFR (mL/min/1.73 m^2^) = 194 × (serum creatinine (mg/dL))^−1.094^ × (age (years))^−0.287^ × 0.739 (for women)) [18]. Because plasma and urinary levels of both NGF and catecholamines were not normally distributed (skewed data), they were Log-transformed to analyze as continuous variables.

Comparisons of continuous variables and categorical variables between the hypertensive and normotensive group were performed using Student's *t* test and the chi-squared test, respectively. As for plasma NGF, the analysis was performed only in 45 cases of the hypertensive group and 46 cases of the normotensive group because of the technical assay problems occurring in the remainders. Group comparisons were also assessed using the standardized partial regression coefficient (*β*) for hypertensive status (hypertension = 1, normotension = 0) with plasma and urinary levels of NGF and those of catecholamines as the dependent variables and variables that were statistically different between the groups (i.e., age and sex) as covariates in multiple regression analysis.

To estimate the associations of NGF status with sympathoadrenal activity, Pearson's correlation coefficients were evaluated between plasma levels of NGF and catecholamines and between urinary levels of NGF and catecholamines. The partial correlation coefficients adjusted for age and gender were also calculated. In addition, the correlations of plasma and urinary levels of NGF as well as those of catecholamines to blood pressure or pulse rate were analyzed. As it has been apparent that NGF can be synthesized and secreted by adipocytes [19, 20], attention was also paid to the associations of NGF status with body mass index in all studied subjects.

All statistical analysis was undertaken using IBM SPSS 25 statistics for Windows (IBM Corp Armonk, NY). A *p* value less than 0.05 was considered statistically significant. Because of no available data in the literature, we could not calculate the number of subjects necessary for an adequately powered statistical analysis prior to the study. However, a two-tailed post hoc power analysis using R Software version 4.1.2 showed that our statistical power to detect a significant difference between the groups with an alpha error of 5% was 99.3% and 80% for plasma nerve growth factor and urinary nerve growth factor, respectively. Thus, the post hoc power analysis revealed that our sample sizes were appropriate for evaluating the intergroup comparison in NGF status.

## 3. Results

### 3.1. Clinical Characteristics

The patients in the hypertensive group were significantly older than the patients in the normotensive group (Table 1). The sex distribution differed significantly between the two groups. Although height was lower in the hypertensive group than in the normotensive group, body weight and body mass index did not differ between the two groups. Due to the study design, blood pressure was significantly higher in the hypertensive group than in the normotensive group. Urinary albumin, serum creatinine, and eGFR did not differ between the hypertensive group and the normotensive group.

### 3.2. Nerve Growth Factor and Catecholamines

Plasma and urinary levels of NGF were significantly greater in the hypertensive group than in the normotensive group (Table 2). Plasma and urinary levels of adrenaline were significantly greater in the hypertensive group than in the normotensive group. Likewise, urinary noradrenaline was significantly elevated in the hypertensive group compared with the normotensive group, although plasma noradrenaline did not differ between the two groups. Most importantly, the abovementioned differences of NGF and catecholamines between the groups were significant even if the confounding factors such as age and sex were adjusted (Table 3). In addition, although plasma NGF was not measured in nearly half of the subjects in either group in the present study, the demographic and clinical data did not differ between the plasma NGF-measured subjects and plasma NGF-unmeasured subjects in the normotensive group (46 versus 35 cases) or the hypertensive group (45 versus 38 cases) (data not shown).

### 3.3. Relationships between NGF and Catecholamines

**Figure 1:**
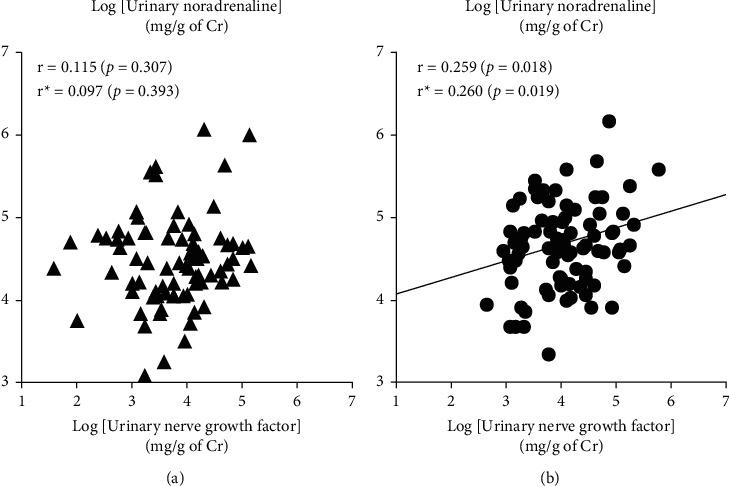
Relationships between the urinary nerve growth factor and urinary noradrenaline in the normotensives (▲, (a)) and the primary hypertensives (●, (b)). Pearson correlation coefficients (*r*), partial correlation coefficients (*r*^*∗*^), and *p* values are shown. The urinary nerve growth factor correlated positively with urinary noradrenaline in the primary hypertensives, but not in the normotensives.

**Figure 2:**
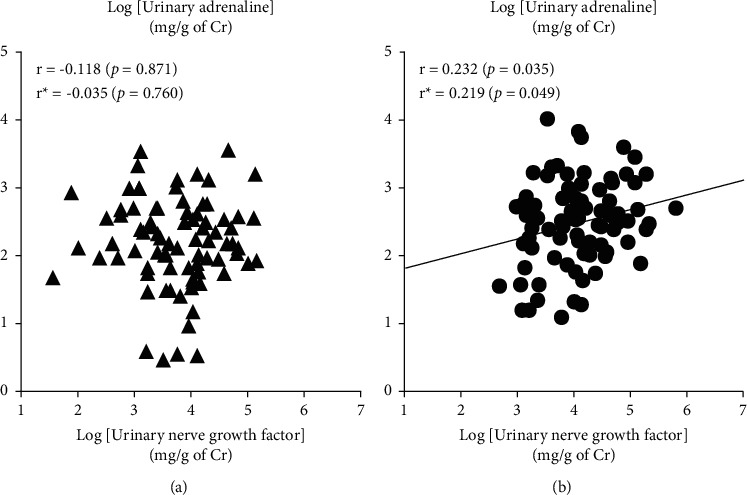
Relationships between the urinary nerve growth factor and urinary adrenaline in the normotensives (▲, (a)) and the primary hypertensives (●, (b)). Pearson correlation coefficients (*r*), partial correlation coefficients (*r*^*∗*^), and *p* values are shown. The urinary nerve growth factor correlated positively with urinary adrenaline in the primary hypertensives, but not in the normotensives.

Plasma NGF did not correlate with plasma noradrenaline or plasma adrenaline in all studied subjects, hypertensive subjects, or normotensive subjects (Table 4). On the other hand, in all studied subjects, urinary NGF significantly correlated positively with urinary noradrenaline but not urinary adrenaline (Table 4). Such positive association between NGF and catecholamines was more apparent and remarkable in the hypertensive group than in the normotensive group. That is, urinary NGF significantly correlated positively with both urinary noradrenaline and urinary adrenaline in the hypertensive group, but not in the normotensive group (Figures 1 and 2). Partial correlation analyses also confirmed that the abovementioned correlations were not affected by covariates of age and gender (Table 5). On the contrary, plasma and urinary levels of NGF as well as those of catecholamines did not associate with blood pressure or pulse rate in either group (data not shown). In addition, plasma NGF but not urinary NGF significantly correlated positively with BMI in all studied subjects (*r* = 0.209, *p*=0.046 for plasma NGF; *r* = 0.005, *p*=0.951 for urinary NGF).

## 4. Discussion

To the best of our knowledge, the present study was the first one to ascertain NGF status and its association with sympathoadrenal activity in human primary hypertension. The major findings of the present study are as follows: First, plasma and urinary levels of NGF as well as those of catecholamines were elevated in the hypertensive group compared with the normotensive group except for plasma noradrenaline. Second, despite no correlations between plasma levels of NGF and catecholamines in both groups, positive relationships were found between urinary levels of NGF and catecholamines in the hypertensive group but not in the normotensive group. Thus, the enhancements of both NGF status and sympathoadrenal activity and the positive relationships between them were characteristic phenomena for primary hypertension. Accordingly, our data indicated that in primary hypertension, NGF status could be enhanced and subsequent NGF-induced sympathoadrenal activation might occur in proportion to the degree of NGF status. In addition, sympathetic neural activation could be possibly regulated more preferentially or profoundly by NGF in the kidneys compared with the other sympathetic innervated organs, because the associations between NGF and catecholamines were confined to their urinary values. This thesis is also supported by the previous experimental data showing the overproduction of NGF and its role on sympathetic overactivity in hypertensive animals [11–16]. In contrast, there were no apparent findings suggesting that NGF could affect systemic hemodynamics directly or through its nerve-mediated effects, because plasma and urinary levels of NGF as well as those of catecholamines did not associate with any systemic hemodynamic indices in either group.

Since NGF was discovered in the adipose tissue, adipose tissue-derived NGF has been presumed to be involved in obese and obese-related disorders [19, 20]. In the present study, plasma NGF but not urinary NGF correlated positively with BMI in all studied subjects. This phenomenon was consistent with the observations reported by Bulló et al. [19] that plasma NGF and NGF mRNA in the adipose tissue were higher in obese women than in normal-weight women. However, it seems unlikely that the intergroup difference in NGF status could be attributed to those in adipose tissue-derived NGF, because both groups were nearly equal in BMI.

### 4.1. The Possible Mechanisms Leading to Enhanced NGF Status in Primary Hypertension

The synthesis and release of NGF can be regulated by a variety of factors in the sympathetic innervated organs or tissues [21, 22]. Although the precise causes of abnormal NGF status in primary hypertension cannot be fully clarified in the present study, several mechanisms could be proposed as follows: First, catecholamines, neurotransmitters released from sympathetic nerve endings, have been demonstrated to modulate NGF synthesis in vascular smooth muscle and cardiomyocytes in both in vitro and in vivo experiments [21–23]. This indicates the existence of some feedback regulation of NGF synthesis by sympathetic neural input into NGF-producing organs [21]. In addition, cultured vascular smooth muscle cells increase NGF secretion in response to alpha-adrenergic agonists, whereas their secretion is inhibited by beta-adrenergic agonists [23]. Therefore, a positive feedback regulation of NGF synthesis by sympathetic influences could be formed by the activation of alpha-adrenergic receptors on the NGF-producing organs. Conversely, a negative feedback regulation could be made by the activation of beta-adrenergic receptors on those organs. Furthermore, it has been shown that the blunted beta-receptor responsiveness and the normal or enhanced alpha-receptor responsiveness occur in the peripheral circulation in primary hypertension [3, 24]. Accordingly, it might be hypothesized that in primary hypertension, the feedback regulation of NGF synthesis by sympathetic influences could be more positive due to the greater alpha-receptor activation. This notion might explain the positive relationship between NGF and catecholamines found in primary hypertension. Second, the renin-angiotensin system might be considered as another modulator for NGF production in primary hypertension. Indeed, angiotensin II is known to stimulate NGF synthesis in cultured vascular smooth muscle cells [21, 23]. In SHR characterized by enhanced NGF synthesis, blockade of angiotensin II synthesis by angiotensin-converting enzyme inhibition has also been shown to decrease renal NGF mRNA together with blood pressure reduction [25]. Of course, it cannot be denied that the difference of NGF status between primary hypertensives and normotensives could be due to their genetic differences in NGF gene and/or NGF gene transcription, as reported in SHR and normotensive controls [26].

### 4.2. Study Limitations

The present study has several limitations. First, the causal relationship between enhanced NGF status and sympathoadrenal hyperactivity in primary hypertension cannot be deduced from our study, because this study was a cross-sectional study. Second, it may be problematic that plasma and urinary levels of catecholamines were used as indicators for sympathetic nerve activity. Indeed, plasma noradrenaline is influenced by neuronal or extraneuronal removal of noradrenaline other than release of noradrenaline from the sympathetic nerve endings into circulation [27]. Antecubital venous samples for the measurements of catecholamines represent the venous drainage from the forearm, but not from the other highly sympathetic innervated organs such as the kidneys and heart [28]. Venous adrenaline is low due to uptake of approximately 50% of adrenaline in arterial blood [28]. Urinary catecholamines could be affected by renal metabolism and renal function, although all studied subjects had normal glomerular function. Therefore, muscle sympathetic neuronal activity or norepinephrine spillover is a more desirable and preferred tool for estimating autonomic nerve activity correctly [27, 29]. However, because those measurements were complex and invasive, lots of clinical practices like the present study have used catecholamines for assessing sympathoadrenal activity [30, 31]. Third, the statistic power might be weak in the analysis of plasma NGF, because plasma NGF was not measured in nearly half of the subjects in both groups. That being said, because the demographic and clinical characteristics did not differ between the plasma NGF-measured subjects and plasma NGF-unmeasured subjects in each group, such selection-related influences on plasma NGF might be ignored in this study. Further studies using a larger number of subjects are needed to validate our data of plasma NGF. And finally, little has been known about the origins of circulating NGF and the process of NGF from its synthesis to release into the blood stream [32]. It was also not apparent whether urinary NGF could represent NGF filtered from blood, renal-synthesized NGF, or both. Further studies should be performed to establish the usefulness of plasma and urinary levels of NGF as surrogates for NGF status.

Although limited for these reasons, the present study showed that the enhanced NGF status and subsequent NGF-induced sympathoadrenal overactivity could occur in primary hypertension.

## 5. Perspectives

Although more studies are warranted to solve the issues listed as study limitations, the clinical implication of this study is that enhanced NGF status could be possibly postulated as one of the causes of sympathetic overactivity in primary hypertension. In addition, our results suggest that NGF modulation might be a pharmacological target for interventions in sympathetic nerve hyperactivity which could promote hypertension and hypertension-related cardiovascular structural changes.

## Figures and Tables

**Table 1:** Demographic variables and clinical characteristics in the hypertensive group and the normotensive group.

Variables	Normotensive group	Hypertensive group	*p* value
Number	81	83	
Age (years)	51 ± 14	56 ± 10	0.017
Gender (male/female)	64/17	53/30	0.032
Height (cm)	168 ± 8	163 ± 10	0.001
Body weight (kg)	68 ± 11	66 ± 15	0.219
Body mass index (kg/m^2^)	24.2 ± 3.2	24.5 ± 3.5	0.530
Systolic blood pressure (mmHg)	125 ± 11	159 ± 18	<0.001
Diastolic blood pressure (mmHg)	77 ± 9	91 ± 10	<0.001
Pulse rate (beats/minutes)	65 ± 11	65 ± 10	0.980
Serum creatinine (mg/dL)	0.81 ± 0.19	0.78 ± 0.19	0.266
Urinary albumin (mg/g of Cr)	15 ± 44	21 ± 29	0.258
eGFR (mL/min/1.73 m^2^)	80 ± 19	76 ± 17	0.101

eGFR indicates estimated glomerular filtration rate. Values are mean ± SD or numbers. Comparisons between groups were made by Student's *t* test or the chi-squared test.

**Table 2:** Plasma and urinary levels of the nerve growth factor and those of catecholamines in the hypertensive group and the normotensive group.

Variables	Normotensive group	Hypertensive group	*p* value
Plasma nerve growth factor (pg/mL)	168 ± 188	311 ± 158	
Log [plasma nerve growth factor] (pg/mL)	4.37 ± 1.42	5.56 ± 0.75	<0.001
Urinary nerve growth factor (ng/g of Cr)	54.5 ± 38.8	72.7 ± 54.0	
Log [urinary nerve growth factor] (ng/g of Cr)	3.74 ± 0.76	4.06 ± 0.67	0.006
Plasma noradrenaline (pg/mL)	228 ± 114	242 ± 93	
Log [plasma noradrenaline] (pg/mL)	5.31 ± 0.51	5.42 ± 0.39	0.138
Urinary noradrenaline (*μ*g/g of Cr)	102 ± 70	125 ± 72	
Log [urinary noradrenaline] (*μ*g/g of Cr)	4.47 ± 0.54	4.69 ± 0.52	0.007
Plasma adrenaline (pg/mL)	23 ± 22	32 ± 30	
Log [plasma adrenaline] (pg/mL)	2.94 ± 0.59	3.20 ± 0.74	0.022
Urinary adrenaline (*μ*g/g of Cr)	11 ± 7	15 ± 9	
Log [urinary adrenaline] (*μ*g/g of Cr)	2.19 ± 0.66	2.49 ± 0.63	0.004

Values are mean ± SD. Comparisons between groups were made by Student's *t* test.

**Table 3:** Multiple regression analysis adjusted for age and gender.

Dependent variable	B	95% confidence interval		*p* value	*R* ^2^
		Lower bound	Upper bound		
Log [plasma nerve growth factor] (pg/mL)	0.366	0.443	1.418	<0.001	0.293
Log [urinary nerve growth factor] (ng/g of Cr)	0.198	0.061	0.516	0.013	0.053
Log [plasma noradrenaline ] (pg/mL)	0.067	−0.084	0.205	0.411	0.089
Log [urinary noradrenaline] (*μ*g/g of Cr)	0.145	0.03	0.310	0.046	0.188
Log [plasma adrenaline] (pg/mL)	0.180	0.026	0.465	0.029	0.066
Log [urinary adrenaline] (*μ*g/g of Cr)]	0.188	0.046	0.448	0.017	0.086

eGFR indicates estimated glomerular filtration rate. *β* means the standardized partial regression coefficient (*β*) for groups (hypertension = 1, normotension = 0) in multiple regression analysis using plasma and urinary levels of NGF and those of catecholamines as the dependent variables and age and gender as covariates.

**Table 4:** Pearson correlation coefficients for the relationships between plasma levels of the nerve growth factor and catecholamines and between urinary levels of the nerve growth factor and catecholamines.

	All subjects		Normotensive subjects		Hypertensive subjects	
	*r*	*p*	*r*	*p*	*r*	*p*
*Versus Log [plasma nerve growth factor]*						
Log [plasma adrenaline]	−0.041	0.704	−0.183	0.223	−0.026	0.868
Log [plasma noradrenaline]	0.201	0.057	0.141	0.352	0.141	0.361

*Versus Log [urinary nerve growth factor]*						
Log [urinary adrenaline]	0.142	0.070	−0.018	0.871	0.232	0.035
Log [urinary noradrenaline]	0.219	0.005	0.115	0.307	0.259	0.018

**Table 5:** Partial correlation coefficients for the relationships between plasma levels of the nerve growth factor and catecholamines and between urinary levels of the nerve growth factor and catecholamines.

	All subjects		Normotensive subjects		Hypertensive subjects	
	*r* ^*∗*^	*p*	*r* ^*∗*^	*p*	*r* ^*∗*^	*p*
*Versus Log [plasma nerve growth factor]*						
Log [plasma adrenaline]	−0.078	0.473	−0.138	0.371	−0.069	0.663
Log [plasma noradrenaline]	0.108	0.315	0.048	0.755	0.098	0.537

*Versus Log [urinary nerve growth factor]*						
Log [urinary adrenaline]	0.117	0.138	−0.035	0.760	0.219	0.049
Log [urinary noradrenaline]	0.190	0.016	0.097	0.393	0.260	0.019

*r* ^*∗*^ adjusted for age and gender.

## Data Availability

The data underlying this study are available from the corresponding author on reasonable request.

## Abbreviations

NGF: Nerve growth factor
eGFR: Effective glomerular filtration rate.
